# Sustaining community-based malaria services through stakeholder engagement: lessons from co-creation in northeastern Thailand

**DOI:** 10.1186/s12936-025-05775-y

**Published:** 2026-01-08

**Authors:** Monnaphat Jongdeepaisal, Massaya Sirimattayanant, Orathai Prasert, Suphitsara Maneenet, Paradorn Sopa, Sekson Napchit, Natsipitch Yuenyao, Navarat Singkham, Ittisak Charoensup, Jerdsuda Kanjanasuwan, Thannikar Thongard, Supa-at Asarath, Napat Khirikoekkong, Anne Osterrieder, Rapeephan R. Maude, Christopher Pell, Phaik Yeong Cheah, Richard J. Maude

**Affiliations:** 1 Mahidol-Oxford Tropical Medicine Research Unit, Faculty of Tropical Medicine, Mahidol University, Bangkok, Thailand; 2 Centre for Tropical Medicine and Global Health, Nuffield Department of Medicine, University of Oxford, Oxford, UK; 3 Provincial Health Office, Ubon Ratchathani, Thailand; 4 Raks Thai Foundation, Ubon Ratchathani, Thailand; 5 Ban Nongmek School, Ubon Ratchathani, Thailand; 6 Buntharik Hospital, Ubon Ratchathani, Thailand; 7 Division of Vector-Borne Diseases, Department of Disease Control, Ministry of Public Health, Nonthaburi, Thailand; 8 Division of Infectious Diseases, Department of Medicine, Faculty of Medicine, Ramathibodi Hospital, Bangkok, Thailand; 9 Amsterdam Institute for Global Health and Development, Amsterdam, The Netherlands; 10 Department of Global Health, Amsterdam UMC, Location University of Amsterdam, Amsterdam, The Netherlands

**Keywords:** Malaria, Community health worker, Stakeholder engagement, Co-creation, Thailand

## Abstract

**Introduction:**

In remote communities in the Greater Mekong Subregion, maintaining community-based malaria care is vital to achieving the goal of malaria elimination. This project aimed to collaborate with the community members and implementers of malaria and health programmes, our key stakeholders, to co-create engagement activities that promote the integration of community-based malaria activities that best fit the local context. This article describes the design, implementation and results of this co-creation process, and highlights key learnings and insights, enabling factors and challenges.

**Method:**

In Buntharik district, Ubon Ratchathani province bordering Laos in northeastern Thailand, we adopted a co-creation framework to design and develop iterative and responsive engagement activities, and used a theory of change framework to outline the necessary steps and conditions to achieve the desired co-creation outcomes. Data were recorded in engagement logs, meeting minutes, observation notes, and participant evaluation to measure the results of engagement and extract key learnings from implementation.

**Findings:**

Between April 2023 and June 2024, 36 in-person engagement activities were conducted with approximately 550 participants, to co-create and evaluate locally-owned health education materials—the 2024 Buntharik health calendar—that integrates malaria information with priority local health issues. The co-created calendar offered one potential entry point to maintain malaria awareness in low transmission areas, but future initiatives ideally should secure additional funding sources to maintain the capacity of local health workers. We found that responding to local health concerns and expectations of the communities and stakeholders is the key enabler to co-creation. However, in the context of changing policy, careful thought about the range of scenarios in which co-creation is applied is crucial to plan for sustainability of the integration. Learning from the context of this engagement, new champions could emerge from involving additional stakeholders beyond those involved in malaria service implementation.

**Conclusion:**

Drawing from this stakeholder engagement work, the co-creation process showed strong potential for ensuring the sustainability of community-based health care in the context of declining awareness and advocacy, such as in the case of malaria elimination. The process and its learning can be adopted to any ongoing local collaborative partnership and future participatory action research and community-informed policy considerations.

**Supplementary Information:**

The online version contains supplementary material available at 10.1186/s12936-025-05775-y.

## Background

In the Greater Mekong Subregion (GMS), malaria still remains a public health threat particularly among communities living in rural and remote areas and traveling to border forested areas [1]. Although, since 2014 the subregion has observed a significant decrease of malaria, 91% reduction in cases 95% in mortality, maintaining community-level services remains crucial for detecting residual infections and responding to potential outbreaks [2, 3]. The effectiveness of community-based malaria services depends on availability of human and commodity resources equipped to provide community-based diagnosis and treatment. Village malaria workers (VMWs) are at the heart of providing community-based malaria services in the subregion, with over 35,000 community-level network of workers and volunteers embedded in endemic areas where the highest-risk populations reside [1, 4]. Recruited from local residents, VMWs have been trained to perform malaria testing and treatment as well as to lead or assist in community engagement and target interventions for malaria control and elimination [4]. As malaria rates decline in the GMS, the financial and supervisory support for VMWs, often from external partners, is also declining, particularly in communities where malaria incidence is approaching zero. Discontinuing these services could reduce the uptake of malaria testing and preventive measures, weakening surveillance and increasing the chance of outbreaks.

To intensify elimination efforts, local malaria services are typically managed through a vertical malaria programme—a targeted, disease-specific control approach often operating independently of broader healthcare systems. In areas approaching elimination, these services could instead be integrated into primary care to sustainably engage frontline health workers to deliver malaria services directly [5, 6]. Ideally, local primary care programmes would champion this strategy, assume responsibility for VMWs, and potentially assign them additional roles addressing other local health needs. Local integration is, however, highly context-specific, with primary care facing competing priorities and a limited healthcare workforce that must allocate time and budget to other pressing matters [7–10]. To actualize this integration, question remains about whether VMWs and the community-based malaria services that they provide can be sustained in settings where malaria programmes are downsized.

The active participation of key stakeholders involved in the delivery and uptake of malaria services, especially the VMWs and the communities they serve, is crucial for exploring options for service integration in their contexts. Co-creation offers one approach to facilitating such participation. Embedded in a participatory process, co-creation encourages relevant stakeholders to take part in defining problems and identifying solutions through ongoing dialogue and collaborative action [11]. In public health, potential co-creators often include end-users, such as patients or target populations for interventions, stakeholders or those interested or involved in the health programme, and academics or researchers generating scientific evidence to support programme design [12]. This approach has become popular for solving complex problems in healthcare. However, evidence on co-creation in low-resource settings, particularly in the area of public health, is scarce. Several studies have identified challenges that arise from such initiatives, especially in low-and-middle-income countries (LMICs), around the issues of equity [13, 14] and systemic barriers embedded in implementing contexts [15].

In northeastern region of Thailand, where about 40 malaria cases were recorded in 2024 compared to nearly 15,000 nation-wide [16], researchers and local stakeholders worked together to co-create approaches for integrating malaria services into primary care at the community level. This engagement project is an extension of the Regional Artemisinin-resistance Initiative or RAI3E operational research conducted in Thailand to disseminate the results of the formative research to our local stakeholders namely the community members, VMWs, and frontline health implementers at one of the study sites in Buntharik districk, Ubon Ratchathani Province—a setting with declining malaria cases and shifting health priorities. This article (1) describes the design and implementation of the co-creation process and the results of co-creating a locally-owned information education communication tool which integrates malaria messaging with broader health concerns, and (2) highlights the reflections, key lessons, and insights from the local stakeholders and researchers on the enablers and challenges to integrate community-based malaria services.

### Context of engagement

Buntharik is the province’s largest district by area and is located 92km from the provincial capital, Ubon Ratcjathani, with an estimated population of 68,405 [17]. Residents’ main livelihoods include agricultural activities, such as rice and cassava farming, and working in rubber plantations. In 2023, the province reported an average income per household of 20,969THB (approximately 630USD), which is lower than the national average of 27,352THB [18]. This is not uncommon among communities along international borders in Thailand, where income generating activities are more limited than in other areas [19]. Human and drug trafficking associated with cross-border migration remain pressing social issues in these border communities [20]. Located along the Thai-Lao-Cambodian border, Buntharik recorded the country’s highest malaria incidence rate during an outbreak in 2014 and is in the regional epicenter of artemisinin resistance, with 7,318 cases reported [21]. In this area, forest-going activities carry a high risk of malaria and are associated with the timber trade, patrols in border areas, and cross-border population movement [22, 23]. Although malaria incidence has reduced substantially [24], with only 10 and 6 cases reported in 2023 and 2024 respectively, the area is still prone to outbreaks, as forest-going activities create potential hot spots of ongoing transmission [25].

In Buntharik and elsewhere in Thailand, VMWs, a cadre of trained volunteers also known in Thailand as malaria post workers (MPs), have been tasked to deliver community-based malaria services. These include providing testing with rapid diagnostic testing (RDT), treatment with anti-malarial drugs, and insecticide-treated nets to their patients and the population-at-risk in their communities. These workers are also a key component of the Regional Artemisinin-resistance Initiative (RAI), where the RAI-funded recipients, including the national programmes and civil society organizations (CSOs), expanded malaria services in remote border areas, improve surveillance, and train the workers to provide frontline care. In Thailand, MPs are primarily overseen by the Provincial Health Office (PHO) and work closely with a larger group of village health volunteers (VHVs), primary health units (PCU) or health promotion hospitals (HPH) under primary care programme, to implement malaria control and surveillance activities. In town centres, malaria testing and treatment services are also available at Malaria Clinics (MC) under the vertical malaria programme and district hospitals under the general health system. Under the universal health care (UHC), Thai population receives free healthcare services, including for malaria, at public health facilities. Migrant population can access malaria services provided by MPs, MC, and CSO-operated clinics without fees; at other public health facilities, however, hospital charges may apply for the malaria and other services.

### Mapping the co-creation stakeholders

At the beginning of the engagement project, we approached the key stakeholders to disseminate the formative research results and invited them to take part with the project and throughout the co-creation process (Table 1). In our formative research, the stakeholders were mapped based on their roles in the malaria programme and the primary care programme at different levels of implementation and across organisations. Our primary stakeholders included the MPs in all endemic villages in the district, their supervisors and programme manager at the provincial level, health workers at the HPHs and PCUs at sub-district level, and Buntharik community hospital (a public health facility providing general health services, including treatment for malaria patients, in Buntharik). Additional local representatives were selected to join the collaboration group: a malaria programme lead from Raks Thai Foundation; and Vector-borne Disease Unit and Center leads from the vertical malaria programme, and district dengue surveillance and response team. Representatives and members of the malaria endemic communities within the catchment areas of the selected MPs, including forest goers and their families, village leaders, and VHVs, were also involved directly in the co-creation activities. We engaged closely with the teachers and the students aged 10–12 at Ban Nongmek school in the endemic community, and expanded our network to other local primary schools and pre-school facilities or children development centres in the communities. They were approached using the contact information from the previous studies and individually consulted to confirm their participation. Subsequently, a larger group of government officials, local residents, and the general public, were gradually invited to participate in a series of engagement activities targeting wider stakeholders. These secondary stakeholders included representatives from the local administration office (LAO) at sub-district level, community representatives and members from non-endemic communities in Buntharik. Table 1 summarises the stakeholders, along with their roles in public health and in our co-creation activities.

**Table 1:** Characteristics of our stakeholders and roles in public health and roles in the co-creation process

Stakeholders	Roles in public health	Roles in co-creation process
Primary stakeholders		
Malaria programme workers	Malaria post (MP) workers and malaria programme staff and managers at multiple implementation levels who have direct roles in providing and overseeing malaria services and interventions including testing and treatment, surveillance and response, and control activities. CSO directly implementing malaria programmes was also invited	- Consulted on project design - Invited as collaborator - Participation in brainstorming and dissemination meetings - Co-implementation and participation public campaigns - Direct evaluation of the project
Healthcare workers	Medical professionals, nurses, and primary care unit (PCU) workers in the public health facilities who have direct roles in providing and overseeing primary health care including outreach and facility-based services	
Endemic communities	Members of malaria endemic communities which include at-risk populations such as forest goers and their family members, farmers, village health volunteers (VHV), village chiefs, village committee members, teachers, students	
Secondary stakeholders		
Non-health programme	Government staff and representative of local organisations overseeing public services and welfare	- Participation in dissemination meeting - Participation in public campaigns - Direct and indirect evaluation of the project via an online platform - Recipients of and participation in the online calendar via the “Line” messenger application
Non-endemic communities	Members of communities beyond endemic areas, adult patients at community hospitals, teachers, students	
General population	Members of the public with interest in public health and the project	

The engagement was led by two local female research staff who are residents of Buntharik district (OP and SM), based at a research office in Buntharik hospital, a district hospital in Buntharik town centre. The office is embedded in a clinic delivering services for HIV and tuberculosis patients, referred to as a “special clinic” and separated from the main hospital buildings. The local team has been working closely with a research physician (RRM) and two researchers (MJ and MS). Our previous studies on the assessment of VMW programmes across the Asia–Pacific have suggested that malaria elimination efforts may be sustained through potential expansion of VMW roles beyond malaria across the Asia Pacific [26, 27] and integration of their roles into primary care in Thailand, with the emphasis on tailoring integration to the local context [9]. To explore these options, the team initiated this public engagement initiative, with Buntharik hospital and the Provincial Health Office as key collaborating partners, and with the support of the Bioethics and Engagement team at the Bangkok-based Mahidol-Oxford Tropical Medicine Research Unit (MORU).

### The design of engagement

The project followed a concept of co-creation in community-based health services [28], which outlines a process of knowledge creation between academics and practitioners that could lead to translation of knowledge into practice. The concept has been utilised as an approach to design interventions and knowledge creation by focusing the process around human experience of patients, service users, and practitioners, and the management of their dynamic relationships in complex health systems, resulting in an effective approach in targeting the development of health services for patients and capacity building for providers, or improvement of services for specific population groups [28]. In this project, we adopted the operational definitions and values of co-creation to guide the design of our engagement activities [11]. To facilitate the evaluation, we also adapted and added the concept of co-learning [29]. With this approach, the engagement was conducted in three stages—co-designing, co-producing, and co-learning—whereby the learning took place at every stage of the framework, building on interim outputs from the previous stage (Table 2).

**Table 2:** Conceptual framework for co-creation (adapted from Vargas et al. [11]; Haraldseid-Driftland et al. [29])

Concept	Description and our focus
Co-creation	▪ A collaborative process, where diverse stakeholders work together across all phases—from identifying the problem and developing solutions to implementing and evaluating them ▪ The process involves engaging relevant stakeholders who actively provide information interact across multiple channels, and create value throughout the process with possible outcome of a good or service from the views of diverse stakeholders
Co-design	▪ Stakeholders collaborate identify pre-specified problem. The process involves gaining insights from the stakeholders to create new plans and lead development of activities ▪ In this process, we identify the stakeholders and collaboration channels, and define the problem we aim to solve
Co-production	▪ Stakeholders collaboratively act on such solutions with a focus on making the most efficient use of available resources and assets. The process involves listening to users and providers to enhance the value of good or service ▪ In this process, we engage stakeholders to design potential solutions to the defined problem, pilot them, and disseminate
Co-learning	▪ A collaborative learning process by identifying enablers and barriers to achieving the outcomes of co-creation ▪ The process engages stakeholders engage to share learning from implementation of activities to improve process and quality of good or service ▪ In this process, we integrate learning with our stakeholders throughout the evaluation and assessment of the piloted solutions and the co-creation process

### The engagement process

**Fig. 1:**
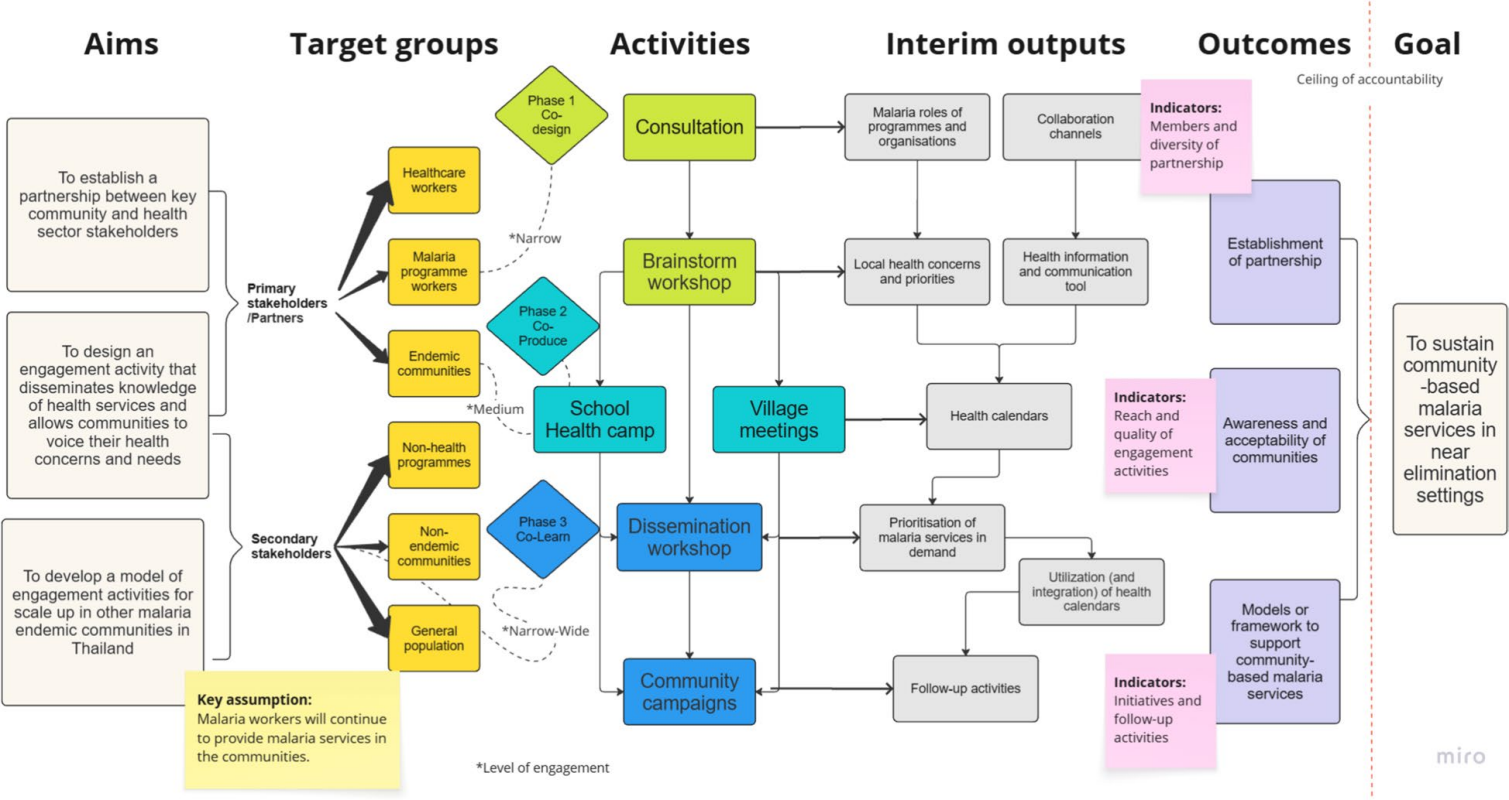
The design and process of engagement activities

In the co-design phase (Fig. 1) we identified and consulted with our primary stakeholders on the design of the engagement activities through consultation meetings and brainstorming workshops. The interim outputs were the identification of MP’s malaria and non-malaria roles, local health concerns and priorities, and potential engagement channels (specifically, target stakeholders and their locations within the communities). These interim outputs outlined the entry point to integrate malaria activities into primary care, by leveraging the embedded roles of MPs as community-based health communicators to utilise Information, Education, and Communication (IEC) tools integrating information on malaria and other local health priorities to raise awareness and promote preventative behaviours in their communities. Past malaria health education materials used or produced by the national malaria programme tended to focus on a single disease and were used to inform populations-at-risk when they sought care with MPs, or when MPs performed active screening within the communities. Considering the seasonal epidemiology of diseases and their associated risks with local socioeconomic activities, IEC tools integrating information about multiple diseases can help both at-risk populations and a wider group of community members stay informed on health issues they are at risk of and how to prevent and recognise them at critical times, for example, prior to their forest travel. From initial consultation, this entry point also presented an opportunity to mobilise local resources for malaria from local authorities interested in using or adapting the tool further with other existing disease programmes. The engagement team and our local stakeholders, hence, decided on compiling and delivering health information in the form of a yearly calendar. Inputs on seasonality and local risks were gathered from the MPs and health workers in the brainstorm workshops, and the community members in the village meetings.

In the co-producing phase, local communities, specifically those within the catchment areas of MPs, were invited to reevaluate the list of identified health priorities and the IEC template in order to ensure the comprehensiveness and usability of the calendar. This was conducted through townhall meetings comprising of village chiefs, members of the village committee, VHVs, and adult members of the communities. We subsequently co-hosted a one-day school health camp at the aforementioned local public primary school to co-produce IEC materials on malaria and dengue. Both of these were highly prioritised for disease surveillance among school children at the time. A local artist was engaged to design and produce the calendar, incorporating inputs from the communities, students and teachers at the school in Buntharik district (for full calendars of the project, please see 10.5281/zenodo.15775272).

**Fig. 2:**
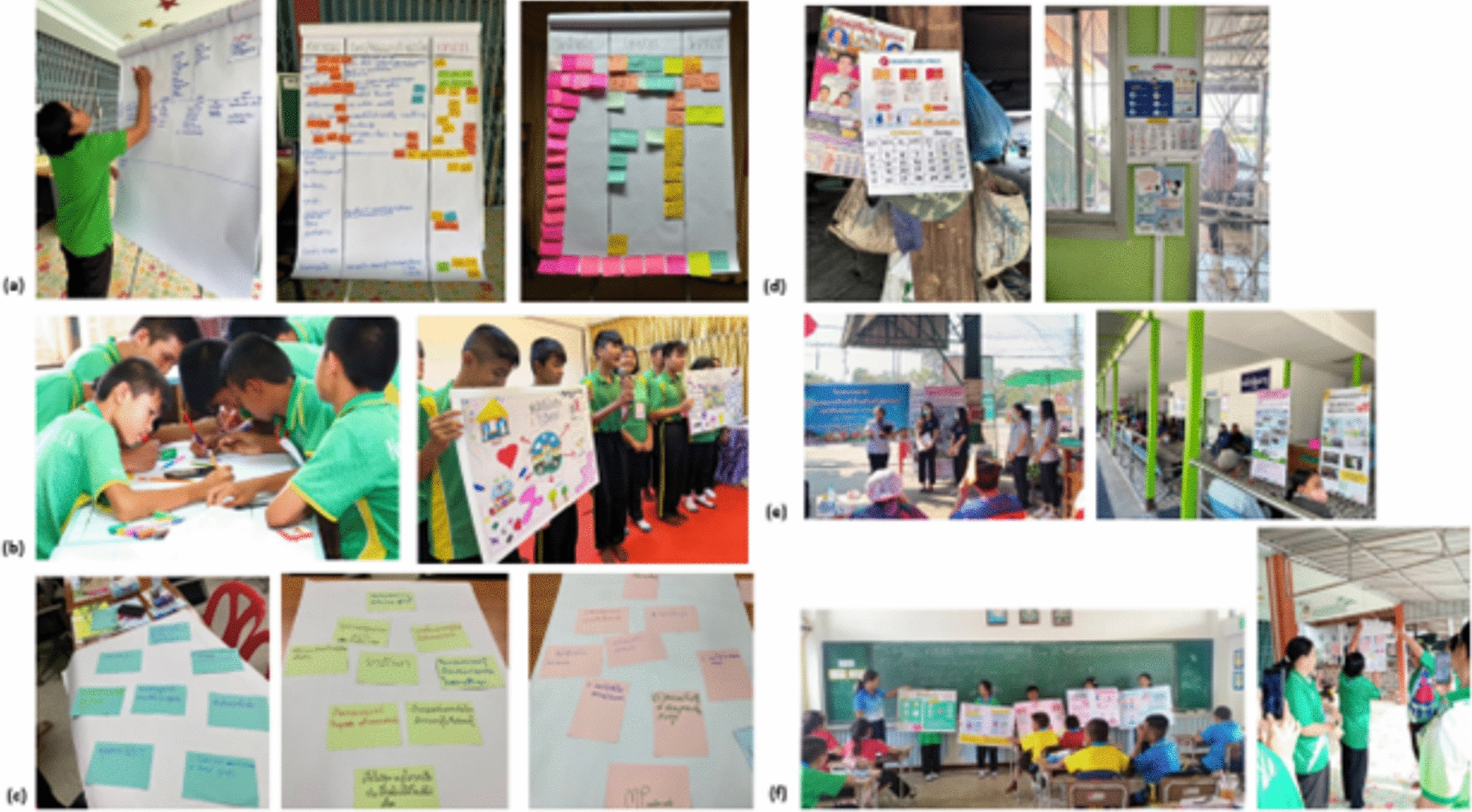
**a–f** Images from co-creation, dissemination and evaluation activities: (**a**) mapping the roles of MPs and identifying local health needs, (**b**) students producing and presenting the co-created IEC materials, (**c**) stakeholders working together to prioritise locally-demanded malaria activities, (**d**) set-up and utilisation of health calendars in a community and health centre, (**e**) public campaigns at various communal spaces and online platform via Line, and (**f**) follow-up use of the IEC tool during health education sessions in classrooms and among MPs and VHVs

In the co-learning phase, we facilitated a series of evaluation activities to extract the learnings and key reflection from the stakeholders and wider community members. The first co-learning activity took place at a one-day dissemination workshop. Here, our key stakeholders in the malaria programme, including MPs and direct implementers of the malaria programme at local and national levels, shared their perceptions and updates on the malaria situation to engage non-malaria programmes on malaria elimination strategy. This was followed by a co-prioritisation activity, where participants identified and prioritised malaria-related activities they perceived should be continued onsite. Printed calendars were also disseminated to be used and shared within their organisations and communities. Through feedback from our co-creation partners, the electronic forms of the calendars were produced and shared online along with monthly health information via a popular and locally used communication platform and the messenger app ‘Line’. We set up a ‘Line’ official account (Line: @961xbdww) to engage with participants beyond our primary stakeholders. Additional activities implemented on the messenger app included online quizzes featuring monthly health information, which helped to continuously expand stakeholder engagement over time. Over a period of 6 months, we tracked engagement with this online platform along with feedback and observations collected by our local team during the follow-up visits in the communities and with the partners (Fig. 2).

### Evaluating the engagement process

Throughout the project, data was recorded in the form of engagement logs, meeting minutes, observation notes, and participant evaluation through questionnaire-based surveys and interviews. During engagement activities, photographs and outputs from groupwork were documented and incorporated in our reflection and debriefing sessions. Throughout the project period, the engagement team met on a weekly basis to plan and review the project. Table 3 describes data sources collected from each activity and throughout the project, incorporating qualitative data derived from observational records, and quantitative data from engagement logs and participant evaluations.

**Table 3:** Data sources from engagement activities

Data sources	Type of data	Activities
Engagement logs	Records of participant characteristics (number, gender, organisations) and context of activities (locations, settings)	All activities
Observation notes	Records of research teams’ observation on the activities, key discussions and questions from participants, enabling factors, and problems or challenges	All activities
Meeting minutes	Written notes from the meetings at various stages of the project including formal and informal meetings with the stakeholders	- Consultation meetings - Brainstorming workshop - Dissemination workshop - Village meetings
Participant evaluation	In-person and online surveys and interviews to collect the level of participation, changes in knowledge and attitudes, and general feedback on selected activities	3 main co-creation activities: - Brainstorming workshop - Dissemination workshop - School health camp

Data was primarily extracted to measure the predetermined indicators and evaluate the extent to which we were able to achieve our outcomes. Subsequently, the team reviewed these evaluation results and extracted key learnings and enablers of successful co-creation activities. This reflective exercise also facilitated our recognition and analysis of the challenges arising from the co-creation process, the contextual health system conditions, and the policy issues at play throughout the project implementation in order to inform subsequent initiatives at site and in other settings.

### Results from the engagement and co-creation activities

**Fig. 3:**
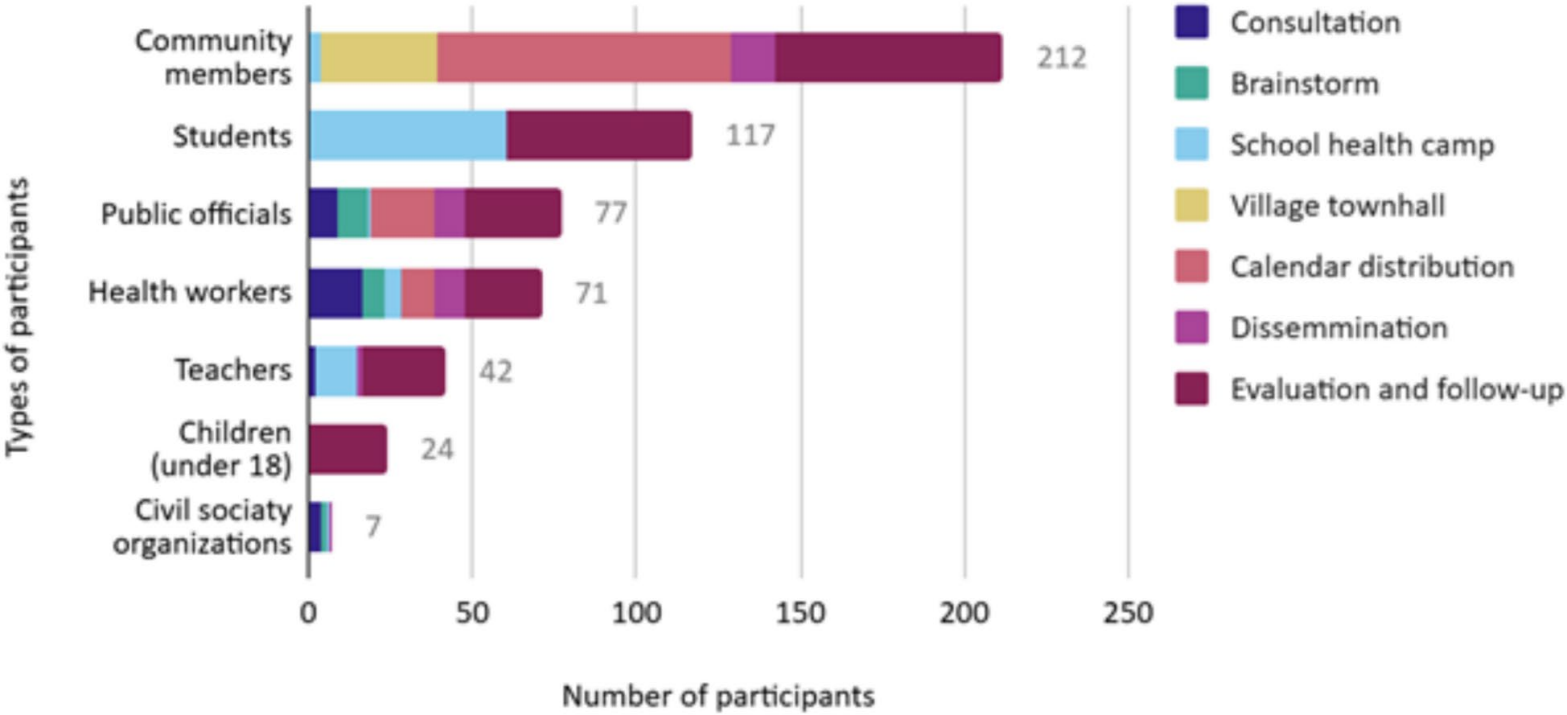
Participant numbers in different engagement activities

In total, we conducted 36 in-person engagement activities with 550 individuals participating in 3 main co-creation activities, 13 consultation meetings, and 20 public campaigns, dissemination and evaluation activities (Fig. 3). Of all participants, 310 were women and 240 men. Our key stakeholders, namely the health workers and public officials involved directly in malaria or relevant health programmes, participated in all the activities. The largest groups of participants were community members and students participating in our public campaigns. In addition, during the dissemination phase, 42 online engagement activities had participation of 139 individuals via the Line messenger app (as of June 2024).

**Fig. 4:**
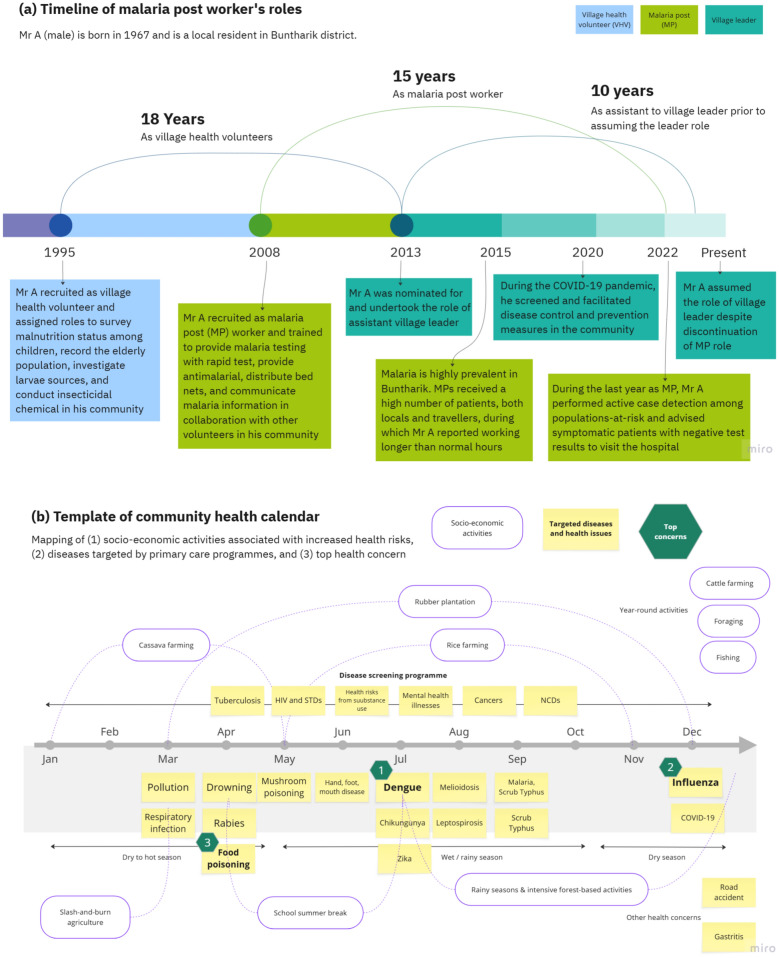

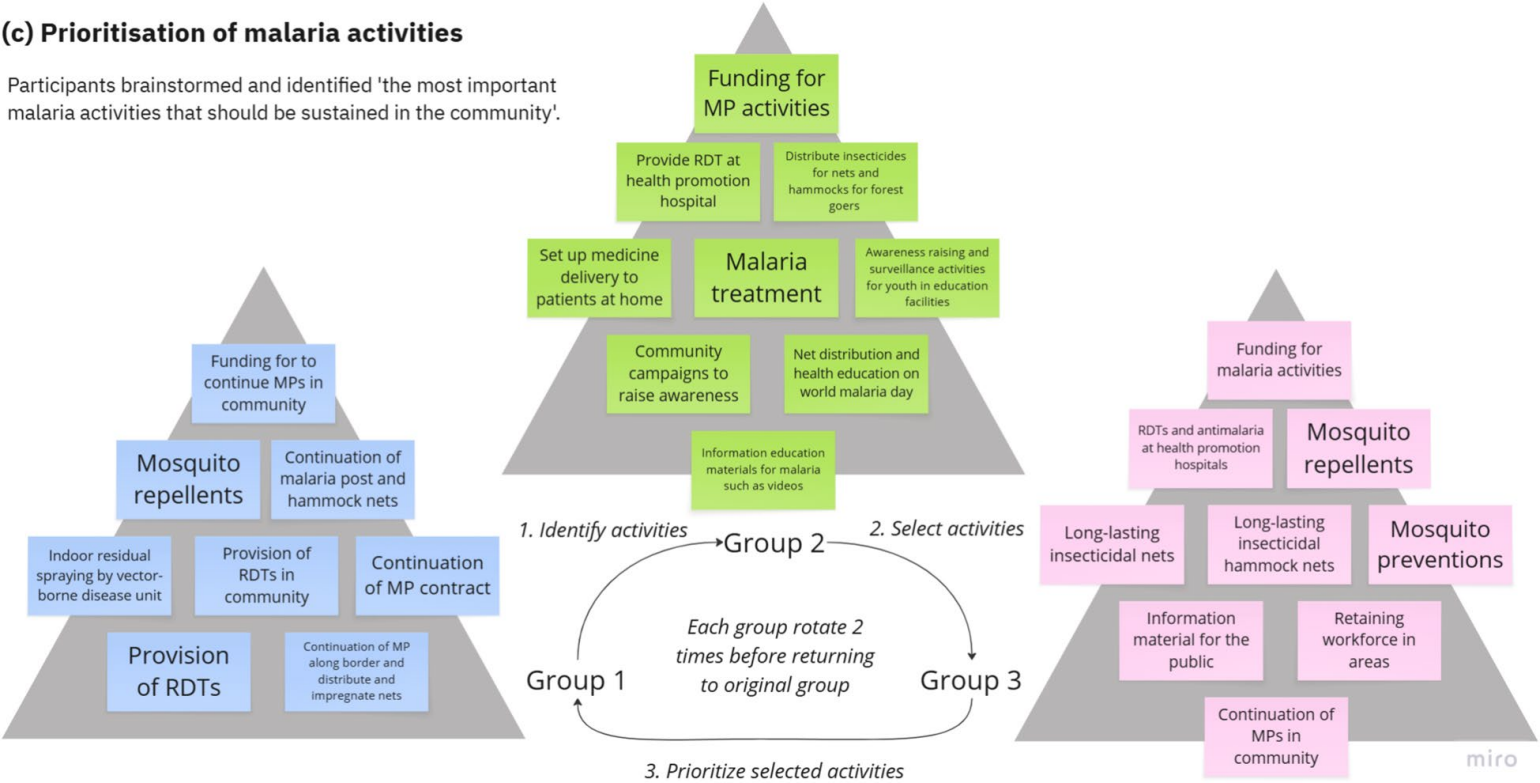
**a–c** Diagrams showing example results from the activities to visualise (**a**) MP life story and roles timeline, (**b**) identification of local health concerns by month, and (**c**) prioritisation of malaria activities among stakeholders during dissemination workshop in order of priorities (high-priority activities are placed at the top); *RDT* rapid diagnostic test, *MP* malaria post worker. Actual results were recorded on paper in Thai

During the brainstorming workshop in the co-design phase, MPs were asked to reflect on the malaria services they provided, and non-malaria roles they performed over the course of their careers and to visualise them in a timeline. This activity underscores a past research finding [9] that a majority of MPs had been recruited from among VHVs, a role many still perform concurrently, linking them with primary care programmes and highlighting their pivotal role as health communicators and bridges to the formal health sector in their communities. An example of a timeline depicting an MP’s roles over time is presented in Fig. 4a. These visual diagrams were presented to wider stakeholders in workshops to highlight the embedded roles MP have to produce a shared understanding in the communities and facilitate the discussion on how to formally support the integration of their malaria roles with use of health education material and other health services in the communities.

The second activity of the brainstorm workshop focused on the identification of local health concerns. Participants were asked to identify health issues and diseases that were critical in the communities, writing down their submissions individually, before placing them on a whiteboard for further discussion and voting. In addition, they were also asked to identify at-risk groups and behaviours for each health concern. Figure 4b presents the visual diagram of an annual timeline with seasonality and socio-economic activities contributing to increased risk of certain health issues and diseases among specific population groups; these inputs were subsequently used to design the health calendar.

The subsequent dissemination workshop in the co-learning phase was designed to engage the participants in brainstorming and co-prioritisation of future of malaria services in Buntharik district. Firstly, participants individually wrote down on cards the three most important malaria services that should be maintained in their communities, shared them within their group, and placed the cards on the table. Each group then rotated to review and discuss another group’s cards before selecting nine cards they perceived as most important. Lastly, groups rotated again, discussed the selected cards, and ranked them by priority. Figure 4c visualises each rotation step and shows the results from the prioritisation among three groups of participants, highlighting the priority to maintain MPs and community-based malaria activities.

Table 4 summarises the outcomes from these activities with a set of indicators, showcasing the extent to which the co-creation process was able to produce the interim outputs and desired outcomes. Enablers and barriers to achieving each outcome were extracted and are summarised here.

**Table 4:** Co-creation outcomes, enabling factors, and barriers

Desired outcomes	Interim outputs	Indicators and results	Enablers and barriers
1. Establishment of partnership between communities and key stakeholders	Malaria roles of programmes and organisations: identification and involvement of programmes and organisations who provide malaria services and primary care services to advocate for community-based malaria care Collaboration channels: establishment of collaborative network beyond malaria-related stakeholders to implement engagement activities	Established working group among 26 key partners: - 3 malaria post workers - 6 primary care and hospital staff - 6 local, 4 provincial, 2 national programme staff - 4 researchers Expanded stakeholders: 16 individuals including village health volunteers, village leaders, and from schools and government offices	We were able to establish and sustain a good level of participation among co-creation partners throughout the duration of project implementation. This network was also expanded to include non-malaria stakeholders; however, discontinuation of MPs at sites and re-assignment of provincial malaria programme managers challenged the sustainability of this partnership
2. Community awareness and acceptability of the designed engagement activities and dissemination of knowledge regarding health services that correspond with local demands	Engagement tools: identification of preferred engagement activities (e.g., meetings, workshops, campaigns, including in-person and online engagement channels) Local health concerns and priorities: identification of health issues that were perceived as priorities by communities and local health providers Health calendar: utilisation and feedback on the IEC materials integrating information about malaria and other local health priorities	Involvement of 550 participants in 36 in-person engagement activities: - 3 primary co-creation activities in each phase - 13 consultation meetings - 20 public campaigns, dissemination and evaluation activities Expanded the dissemination online engaging 139 individuals from 42 online engagement activities in the Line app’s official account	The project leveraged the key stakeholders to organize multiple rounds of engagement activities that reached endemic communities and the general public. Positive feedback on the health priorities incorporated into the health calendars reflected the effectiveness of co-creation in identifying health risks perceived at the local level. Level of engagement among school students and participants in the dissemination meeting could be improved
3. Model or framework to support scaling up	Prioritisation of malaria services in demand: co-identification of malaria services that the local implementers and community representatives, including the working group and expanded stakeholders, perceived as necessary and should be maintained in the communities Follow-up activities: Additional activities or new initiatives taken up by our stakeholders beyond the end of the project	Locally owned health calendar 2024: - 100 calendars distributed; with 36 tracked, observed, and followed-up in-person for feedback (14 among stakeholders and 22 among expanded network) - 2 MPs utilised the calendar as an IEC tool in their VHV roles - 4 schools adapted calendar materials and used them to conduct health education sessions in classrooms	Co-creation activities were able to address power asymmetry in the partnership among volunteers and professionals by using techniques such as anonymity and co-reviewing of results. MPs were able to advocated for their roles among local implementers and national programme staff; however, there were limited follow-up initiatives from the provincial and district health programmes

### Reflection from the stakeholders on the engagement and co-creation activities

In addition to the indicators already presented, we documented participant feedback on the engagement activities, followed up on their use of the calendars, and gathered their perspectives on the future of community-based malaria services. Reflections from the stakeholders focused on the integration of local health concerns and the extent to which the calendars can be utilised to communicate these concerns in the communities. Table 5 summarises the key reflections from the stakeholders and the participants on the engagement activities and the co-created IEC tool.

**Table 5:** Summary of evaluation, feedback and stakeholder reflections regarding the engagement activities and use of the co-created calendar

Question	Feedback	Reflection from stakeholder
How was your experience and participation in co-creation?	- More time allocation for the smaller group activities and series of activities over time could be designed to encourage more participation from the school students and dissemination workshops - More ice-breaking and introduction of participants will provide opportunities for networking among extended groups of stakeholders	*“I think the students will benefit from participating in these activities [dissemination workshop] and apply what they learn when they are back at school.” Key collaborator at school describing her experience from the dissemination workshop and potential to involve students as participants in these activities in the future* *“I like the idea of engaging with the schools but one-time activity might be too limiting and lead to low levels of participation from the students. Two days or more are better for learning activities and provide them with more opportunity to present their work.” Key collaborator at community hospital*
What are your learnings and advice for our project and future health communication and malaria campaigns?	- Forest going is still common among the communities who are familiar with malaria from past experiences, but have minimal concern about the disease - High level of advocacy for dengue and activities related to dengue. Health education sessions or material should focus on a combination of febrile illnesses - Suggestions from key stakeholders to organize a series of meetings (e.g. 2–3 times/year) to share learnings and updates on malaria at community level - Suggestions to create a shared folder for the project to collect and share updated documents regarding malaria information	*“Follow-up activities could be designed in collaboration with the hospital. We can look at clinical case records and suggest the disease or health issues that we should inform the communities about in a timely manner […] This will make them active and more aware of the diseases.” Key collaborator at community hospital* *“Currently if the hospital receives patients with a history of forest travel they will be tested for malaria by microscopy. But we have seen more patients with dengue and experienced delayed treatment for this group. What we need is a faster referral process […] I suggest future health communication focus on febrile illnesses such as leptospirosis, dengue, COVID, influenza, scrub typhus, and malaria.” Key collaborator at community hospital* *“Our work now focuses mostly on dengue surveillance in transmission areas, actually the same areas as we conducted malaria surveillance, but malaria activities are more limited […] Meetings are now mostly hosted by the national programme.” Key collaborator at vector-borne disease unit*
Where are the calendars located?	- Many calendars are placed on support columns in homes and/or on the walls in indoor settings or office spaces - Convenient for use in providing mobile health education and in group settings - Preference for other types of calendar such as desk calendar, especially among facility-based staff - Suggestions from local residents and general public for adapting IEC materials in calendar as larger posters placed around communal spaces	*“I like the calendar. I think I would use it more if they are produced as a desk calendar. We also shared the invitation to follow the calendar with the Line groups of village health volunteers and village leaders in our community. The physical one we placed at the area where if patients come to see us and get their blood pressure and weight measurement, they can read it and we inform them about health issues of the month.” Key collaborator at health promotion hospital* *“If it is a desk calendar I would be able to use it when I am tending to patients like pregnant women and non-communicable disease patients while they are sitting and receiving the services. I think what can be added is information about first aid and injury treatment such as insect bites or bee stings. Locals often got bitten when they forage for honey in the forest” Key collaborator at health promotion hospital*
What do you think about the design and function of the calendar?	- The design was well-perceived overall - Suggested to enlarge the infographics, fonts, and size of the calendar - Date grids are most utilised for users, particularly when placed within facility-based spaces - Suggested to add details of public holidays and lunar calendar details, as they are important for most communal activities, especially for Buddhist events - Little attention paid to the background information; many suggested that general public may not be aware of the malaria background	*“The information and the graphics are well designed but if I place the calendar high on the wall or far from the tables in the shop I think people would not be able to read them properly.” Calendar recipient and a member of general public* *“The content about health is very useful. I normally also look for Buddhist Lent days in a calendar but this one does not have information about the Moon’s Phases [such as in lunar calendar] so I could not tell which day is full moon or new moon. [the dates when residents often gather for merit-making activities at temples and organise other celebrations in the communities]” Calendar recipient and a member of general public*
What do you think about the list of local health priorities?	- The most popular content was on mushroom poisoning, dengue, rabies, PM2.5, and RSV - Many interested in children-related health issues such as drowning, and hand, foot, mouth disease - Suggestions to add IEC on diarrhoea, food poisoning, conjunctivitis, first aid and treatment of basic injuries	*“I really like the information about health concerns targeting children like drowning or hand, foot, and mouth disease because we often visit schools to conduct health education. This is helpful because we can use it directly to communicate with students. The volunteers can use them as well.” Key collaborator at health promotion hospital* *“It will be difficult to apply for funding to conduct malaria-only activities because it is not perceived as important enough in the community. Funding requests need to be made in combination with other diseases like dengue […] we recently reported 17 dengue patients and some of them presented severe conditions because they purchased medication by themselves and sought care late. One patient is non-Thai and discouraged to visit the hospital because of the cost. The person also went to MP for malaria test but the test was negative, then went to a private clinic vomiting blood and was only then referred to the hospital […] Activities for malaria should be matched with local problems like this to justify for funding.” Key collaborator at PCU*
How was the calendar used?	- Teachers and MPs used the calendar as a health education tool in classroom and school activities - Appropriate size for outreach health education sessions; but not in a large classroom (with 15–20 students)	*“The calendars received a fair amount of attention from my neighbours, when they come to see me they read and look at the pictures from the activities at the local school. Every Thursday the village health volunteers will meet at my place to conduct our routine household visit for foci investigation (dengue) and I usually inform them then about what health issues we should be looking out for each month” Key collaborator and former MP* *“Dates and Buddhist Lent days are what I looked for most in the calendar. Currently there is interest in mushroom poisoning as it is seasonal. I brought the calendar to the monthly village meeting to inform the communities about the health issues each month. The material is useful and appropriate for when I need to travel and take it with me. The hanging calendar is good because people can still read the text, especially for older people. The desk calendar is not suitable for health education activities.” Key collaborator and former MP* *“We initiated a new project on dengue prevention in schools and referred to the school activity we organized together and the content in the calendar. […] We also shared the information from the calendar among schools, teachers, and parents in our network. Our teachers also used the information provided in the calendar to create quizzes and test questions.” Key collaborator at a local school*

### Key lessons on the integration of community-based malaria services

This section highlights key lessons and insights from stakeholders’ feedback, and researchers’ reflections on the co-creation process. We have conceptualised these into three key considerations for programme implementers looking to implement a sustainable approach to supporting community-based malaria care in endemic communities with low transmission of and priority for the disease. The following reflections depicted the key lessons from the overall implementation and evaluation of the project and the feedback and evaluation from the stakeholders through debrief and end-of-project meetings and consultations are also incorporated in these learnings. Overall, responsiveness to local communities and stakeholders emerged as critical for building trust and maintaining relevance of the engagement activities, particularly in the face of unexpected contextual or operational changes. Co-created and locally grounded health education materials were shown to be essential for sustaining malaria awareness; however, their impact was strongest when complemented by ongoing capacity-building efforts that empowered community actors to translate knowledge into action. Importantly, the integration process also revealed opportunities to cultivate new champions for malaria beyond traditional implementers, as engaging a broader range of stakeholders helped strengthen ownership, and reinforce the long-term sustainability of community-based malaria responses.

1. Responsiveness to local communities and stakeholders is crucial in building trust and sustainability of the engagement despite facing unexpected changes
  In this project, we conceptualise responsiveness as how the project reacts or responds to the needs and concerns of the community and stakeholders we worked with. Responsiveness is crucial in building trust and sustainability of our engagement. During our implementation, we established a network of diverse stakeholders in terms of positions—from volunteers to professional workers directly and indirectly involved in the provision of malaria services. The collaboration facilitated our ability to respond to their expectations on the co-created health material, and formed a shared value of community-based malaria services, as well as a vision for the future of malaria activities (see Fig. 4a–c). However, a discontinuation of MPs at site limited our ability to respond to the transition of malaria services from MPs to primary care in a timely manner. Hence, we found responsiveness to be a recurring challenge when engagement is operating under a changing policy context, requiring adaptive strategies and reflexivity in responding to these changes while managing the community and stakeholders’ expectations. Understanding and planning for unexpected changes, such as in the case the removal of key community-based and programme-based workers, could enable the engagement to better cope with challenges in sustaining the partnership, as well as, to optimally respond to the transition of MP’s community-based services to facility-based services provided by the health centers and hospitals.
2. Co-created integrated health education material is crucial for maintaining malaria awareness but ideally should be followed by capacity building activities
  With the decline of malaria cases and perceived risk of the disease, the importance of the prolonged awareness and acceptability of malaria services among the remote communities, as well as the implementers, cannot be understated. In building the local health calendar as the key output of the co-creation, a consideration was made that the IEC materials for malaria would only be able to maintain this discussion among the stakeholders and enhance their attention when integrated with other local health concerns. This option could strategically pave way for other malaria activities to also be integrated with other public health campaigns that target similar populations-at-risk or diseases. Brainstorming workshops with local health service providers emphasized the need for integrating information and services related to other vector-borne diseases and local illnesses—such as dengue, influenza, and food poisoning—to maximise the awareness campaigns and increase the acceptability of potential service users. Our experience with the calendar co-creation showcased potential for local implementers to integrate such interventions from the bottom-up by responding to the local needs in the communities, conducting health education sessions and raising public awareness about malaria and other local health concerns together. Nevertheless, the project was unable to secure local funding to continue or expand on this strategy and sustain related activities beyond the project’s completion. Future attempts should explore this alongside capacity building activities or interventions; for example, local distribution or provision of long-lasting insecticide treated bed nets and topical repellents as part of an integrated prevention measure with dengue programme as indicated in Fig. 4c.
3. New champions could emerge from involving stakeholders beyond key malaria implementers
  Co-creation is a collaborative process of brainstorming, designing, and learning, and in our case, of approaches to inform community-based malaria care, which naturally would focus the action and attention on the direct implementers of malaria services. Yet, effective integration of these approaches needs to look beyond the usual stakeholders of malaria programmes that are facing challenges of limited resources and competing local mandates. Involvement of new stakeholders could potentially progress the integration of malaria care into the broader community landscape. Following the co-learning activities, local schools and teachers emerged as key actors, taking the initiative further and using the co-created IEC tool to raise awareness of health risks and prevention measures among youth populations. There are several reasons for this active participation. Knowledge and practice of malaria prevention not only benefits the population at risk for malaria, but also those susceptible to other vector-borne diseases in the community. This is particularly the case for dengue control, with at-risk school children as the main target population for local implementers. Leveraging school-specific interventions (such as preventative health education for dengue, flu vaccination, dental care) can help broaden the scale-up of malaria integration effort by involving parents and PCUs responsible for their children or patients, leading to a comprehensive ecosystem of IEC strategy for local health issues.

### Enablers and barriers to bottom-up engagement framework

Throughout the co-creation and learning process, we identified key considerations for local implementers adopting co-creation approaches to sustain community-based malaria care. These include responding to local needs and engaging in communication and advocacy with decision-makers and broader stakeholders. In our case, co-producing IEC materials proved to be a feasible entry point for bottom-up integration. However, various entry points can be adapted to meet local stakeholder needs. For example, participatory research on dengue control has used co-creation to support community mapping, clean-up campaigns, bulk purchasing of prevention tools, and the development of larvicide use guidelines [30]. Such activities—particularly those focused on resource mobilization and capacity building—offer promising models for sustaining co-creation efforts. Based on the observed contextual challenges, future co-creation strategies should also consider securing additional funding to support continued malaria services or integrate them into primary care.

Participatory approaches were most effective when discussions were open and exploratory. Co-creation is most meaningful when diverse stakeholders—from community members to service providers—are actively involved. In our project, co-producing IEC materials that addressed shared local health needs helped gain buy-in across stakeholder groups. Similarly, a co-creation initiative in Ghana developed a board game and brochures to foster inclusion among caregivers and healthcare providers [31]. However, the project faced challenges in terms of securing more support from national-level health programmes to boost its sustainability and expand its impact to a broader audience. While our project encountered limited follow-up from malaria implementers, participant feedback led to strong interest from local schools, which have adapted the IEC materials for classroom and community-based health education. Additionally, ensuring equitable contributions from the outset is essential for meaningful co-creation. Participants should feel confident and empowered to voice their opinions freely in the activities and during interaction with the research team and other participants. Participants should feel confident to express their views openly. In our project, social and professional hierarchies within the community posed challenges. To address this, we used small group brainstorming and later shared results with the wider group and also introduced anonymous input during the dissemination workshop, allowing participants—especially those with supervisory dynamics—to contribute freely. This approach enabled more candid feedback during activities like malaria service prioritisation and created a valuable channel for bottom-up input from frontline workers to programme managers.

Through this engagement project, we have made a case for a bottom-up approach to advance malaria service integration. As previously mentioned, integration is complex and by no means a novel issue; integration of vertical disease programmes has been heavily discussed, and recently restated with continuing advocacy for universal health coverage [32] and health for all policies [33]. Recent recommendations on the integration of service delivery for malaria have argued for more research and implementation with the communities to co-create and foster ownership of the strategies which linked malaria services or integrated them with existing services that are preferable or more highly valued among the beneficiary communities [34]. It also proposed to reframe malaria as an equity issue and underscore the priority for disease elimination and intersectionality of malaria risks with other health concerns. Our project linked local health needs with increased malaria risk, highlighting how social determinants shape disease burden in vulnerable communities, which offered a practical example of reframing health issues through evidence-based insights.

Sustaining community motivation is crucial for malaria elimination efforts, and maintaining the participation for such efforts is a challenging endeavour when malaria is not a perceived priority health concern for local authorities or in community members’ daily lives. Rather than trying to solely promote malaria awareness, our project fostered a bottom-up engagement by addressing topics that directly impacted the community. This required the engagement team to be reflexive and adaptive to their project’s stakeholders. It is also crucial for researchers that are embedding such component into their work to understand the sociocultural environment and motivations of the community to participate in the health interventions [35–37]. Recent studies have showcased such engagement outcomes by measuring their coverage and costs to provide evidence of outcomes related to health and feasibility [38, 39], evaluate the public’s motivations to participate in citizen science project for malaria control [40], and co-create tailored trial implementation based on local knowledge and community-set goal [41]. Unfortunately, we could not measure the uptake of community-based malaria services provided by the MPs involved in our project because their role was discontinued, despite receiving feedback from the MPs that their patients and clients still visited them for their services. We attempted to capture the quality and reach of the project by defining outcome indicators through the established partnership, awareness and acceptability, and tracking of follow-up activities. These evaluations were a step towards measuring the extent to which the project had supported a bottom-up approach to co-creating sustainable malaria elimination efforts. By measuring the extent to which the community has been engaged with locally-led malaria activities that have taken place in their communities, the evaluation will further explore an optimal and context-appropriate approach that ensures the engagement’s retention [42].

## Strengths and limitations

This engagement project built on the formative research, which allowed the research team to identify an extensive network of key stakeholders and built from a dissemination to participatory engagement project. Adopting the co-creation approach has provided us the flexibility and adaptability to tailor the engagement approach to best fit the feedback from the stakeholders and co-create IEC materials that reflected the realities of the communities affected by malaria. However, we acknowledged the power asymmetry from embedded social and professional norms and structures among our stakeholders, and between the stakeholders and the engagement team. To minimize the asymmetry, multiple series of evaluation and feedback sessions were conducted by the local team who belong to the community and have ongoing working relationships with the stakeholders. Tailoring these activities to varied groups of participants and using anonymity of inputs at several stages of co-creation also ensured a fair contribution of the stakeholders.

## Conclusion

The result of this engagement illustrates the practical application of stakeholder engagement in near elimination context and offers a framework for engagement that is responsive to both community and policy-level dynamics. The co-creation approach has strong potential for ensuring the sustainability of community-based health care in the context of declining awareness and advocacy of vertical disease programmes, such as in the case for malaria elimination. The process promoted local collaborative partnerships to co-create integrated community-informed health IEC materials aiming to raise awareness of malaria and various local health concerns, as well as maintain the roles of the key malaria workers as community mobilisers and the first point of contact for patients in their communities. Future attempts to sustain community-based malaria services should explore new opportunities to integrate local health needs into existing malaria interventions, or vice versa, and consider leveraging locally-sourced funding and leadership as an entry point to advocate and initiate such integration.

## Supplementary Information

Supplementary material 1.

## Data Availability

Data is provided within the manuscript or supplementary information files.

## Abbreviations

CSO: Civil society organization
DVBD: Division of vector-borne diseases
GMS: Greater Mekong Subregion
IEC: Information education communication
LAO: Local administrative organization
LMIC: Low- and middle-income countries
HPH: Health promotion hospital
MP: Malaria post or malaria post worker
PCU: Primary care unit
PHO: Provincial health office
RAI: Regional Artemisinin-resistance Initiative
RDT: Rapid diagnostic test
UHC: Universal health coverage
VBDC: Vector-borne disease centre
VBDU: Vector-borne disease unit
VHV: Village health volunteer
VMW: Village malaria worker
