# Supplementary material for: Sustaining community-based malaria services through stakeholder engagement: lessons from co-creation in northeastern Thailand
Source: Malar J. 2026 Jan 8;25:82. doi: 10.1186/s12936-025-05775-y (PMC13007345; doi:10.1186/s12936-025-05775-y)
Supplement: Supplementary file 1 — Supplementary material 1. [file 12936_2025_5775_MOESM1_ESM.docx]

**Supplementary file 1: Plain English Summary and Thai Abstract**

**Plain English Summary**

In remote communities of the Greater Mekong Subregion, making sure that malaria care in the endemic communities is available for people who are at risk is crucial to eliminating the disease. We worked closely with local communities and health program staff to combine malaria activities with other local health priorities. The project took place in Buntharik district, Ubon Ratchathani province in northeast Thailand. From April 2023 to June 2024, the team carried out 36 in-person engagement activities involving around 550 participants. Together, we created and assessed a health education calendar for 2024 that includes both malaria messages and information on locally-raised health concerns. This community-based health calendar helped keep the communities aware of common health concerns when they are most at risk, including malaria, even though the cases are now low. However, we found that keeping community-based activities going will need more funding and capacity supports in the future. What made the process work well was listening to what local communities care about, and adjusting activities to fit those needs. The project also showed that new leaders of malaria activities can emerge when more people—beyond just malaria programmes—get involved. In summary, this stakeholder engagement approach holds promise for keeping health services strong in communities, especially where diseases like malaria are no longer seen as urgent, and could also guide future malaria research and programs.

**บทคัดย่อ (ภาษาไทย)**

**บทนำ** ในประเทศแถบอนุภูมิภาคลุ่มแม่น้ำโขง การคงไว้ซึ่งการตรวจและรักษาโรคมาลาเรียในชุมชนที่ห่างไกลถือเป็นมาตรการสำคัญต่อการบรรลุเป้าหมายในการกำจัดโรคมาลาเรีย โครงการวิจัยนี้มีเป้าหมายเพื่อร่วมมือกับสมาชิกในชุมชนและผู้ดำเนินงานโครงการมาลาเรียและด้านสาธารณสุขในการออกแบบแนวทางและกิจกรรมที่จะสนับสนุนให้เกิดการบูรณาการงานมาลาเรียให้เหมาะสมกับบริบทชุมชนของอำเภอบุณฑริก จังหวัดอุบลราชธานี ซึ่งตั้งอยู่ในภาคตะวันออกเฉียงเหนือของประเทศไทยและมีชายแดนติดกับประเทศลาว บทความนี้นำเสนอแนวทางการออกแบบ การดำเนินงาน และผลลัพธ์ของกระบวนการร่วมสร้างสรรค์โดยการมีส่วนร่วมระหว่างผู้มีส่วนได้ส่วนเสียในโครงการ (co-creation) พร้อมทั้งถอดบทเรียน ปัจจัยส่งเสริม และความท้าทายที่สำคัญที่ส่งผลต่อกิจกรรมการบูรณาการงานมาลาเรียในชุมชน

**วิธีวิจัย** โครงการได้นำกรอบแนวคิดการร่วมสร้างสรรค์มาใช้ในการออกแบบและพัฒนากิจกรรมการมีส่วนร่วมที่ส่งเสริมให้เกิดการเรียนรู้ระหว่างผู้วิจัยและชุมชนตลอดการดำเนินกิจกรรม ผนวกกับกรอบแนวคิดทฤษฎีการเปลี่ยนแปลง (Theory of Change) เพื่อวางแผนขั้นตอนการดำเนินกิจกรรมให้เป็นไปตามผลลัพธ์ที่คาดหวัง โดยรวบรวมแหล่งข้อมูลจากบันทึกกิจกรรมการมีส่วนร่วม รายงานการประชุม การสังเกตการณ์ และผลการประเมินกิจกรรมจากผู้เข้าร่วมกิจกรรมและผู้มีส่วนได้ส่วนเสียสำคัญของโครงการ เพื่อประเมินและติดตามผลลัพธ์ของการมีส่วนร่วมและถอดบทเรียนจากโครงการ

**ผลการศึกษา** โครงการดำเนินกิจกรรมการมีส่วนร่วมในชุมชนทั้งสิ้น 36 ครั้งระหว่างเดือนเมษายน 2566 – มิถุนายน 2567 โดยมีผู้เข้าร่วมโดยประมาณ 550 คน เพื่อร่วมกันออกแบบ ติดตาม และประเมินผลการใช้ “ปฏิทินสุขภาพบุณฑริก พ.ศ. 2567” ในฐานะสื่อให้ความรู้ที่บูรณาการข้อมูลความรู้ด้านมาลาเรียเข้ากับประเด็นสุขภาพท้องถิ่นที่สำคัญ เราพบว่าการออกแบบสื่อความรู้ร่วมกันนี้เป็นแนวทางหนึ่งที่จะริเริ่มให้ชุมชนยังคงไว้ซึ่งความตระหนักเกี่ยวกับโรคไข้มาลาเรียในชุนชนที่การแพร่ระบาดต่ำได้ นอกจากนี้ เราพบด้วยว่าการตอบสนองต่อปัญหาสุขภาพและความคาดหวังของชุมชนและผู้มีส่วนได้ส่วนเสียแต่ละกลุ่มเป็นปัจจัยส่งเสริมหลักในกระบวนการร่วมสร้างสรรค์ และการพิจารณาอย่างรอบคอบถึงบริบทความเปลี่ยนแปลงเชิงนโยบายที่อาจเกิดขึ้นเป็นปัจจัยส่งเสริมที่สำคัญต่อการวางแผนโครงการสุขภาพเพื่อเสริมความยั่งยืนอย่างบูรณาการ จากการถอดบทเรียนของโครงการ เราพบว่านอกเหนือจากผู้ที่มีบทบาทโดยตรงด้านการบริการมาลาเรียในชุมชน กระบวนการร่วมสร้างสรรค์โดยการมีส่วนร่วมระหว่างผู้มีส่วนได้ส่วนเสียกลุ่มต่างๆ ในชุมชน สามารถจุดประกายการบูรณาการงานมาลาเรียในชุมชนไปยังคนอื่นๆ ในชุมชนและเสริมความอยากมีส่วนร่วมในการร่วมกันปกป้องชุมชนของตัวเอง เช่น อาสาสมัครสาธารณสุขและคุณครู และนักเรียนในโรงเรียนที่ตั้งอยู่ในพื้นที่เสี่ยง หากมีโครงการริเริ่มอื่นในอนาคต ผู้วิจัยหรือผู้ดำเนินโครงการควรพิจารณาจัดหาทุนและวางแผนกิจกรรมที่เสริมทักษะความรู้และเสริมสร้างศักยภาพให้กับบุคลากรสาธารณสุขในการบูรณาการงานมาลาเรียร่วมกับงานสาธารณสุขอื่นๆ ในชุมชน รวมถึงการร่วมกันผลิตสื่อให้ความรู้ด้านสุขภาพร่วมกับคนในชุมชนด้วย

**สรุป** จากกรณีศึกษาและตัวอย่างของโครงการงานกำจัดโรคไข้มาลาเรียนี้ สรุปได้ว่ากระบวนการสร้างสรรค์เพื่อร่วมกันเพื่อผลิตปฏิทินสุขภาพชุมชนเป็นช่องทางหนึ่งในการเสริมศักยภาพและความยั่งยืนเพื่อให้เกิดการดูแลตัวเองในชุมชนได้ในสถานการณ์ที่ความตระหนักรู้และการรณรงค์ทางสุขภาพเริ่มถดถอยลง รวมถึงตัวอย่างและบทเรียนของโครงการสามารถนำมาใช้เป็นแนวทางในอนาคตในการวิจัยเชิงปฏิบัติแบบมีส่วนร่วมและเพื่อการขับเคลื่อนการเปลี่ยนแปลงเชิงนโยบายด้านงานมาลาเรียและงานสาธารณสุขท้องถิ่นโดยชุมชนต่อไป
